# Bifunctional non-noble metal oxide nanoparticle electrocatalysts through lithium-induced conversion for overall water splitting

**DOI:** 10.1038/ncomms8261

**Published:** 2015-06-23

**Authors:** Haotian Wang, Hyun-Wook Lee, Yong Deng, Zhiyi Lu, Po-Chun Hsu, Yayuan Liu, Dingchang Lin, Yi Cui

**Affiliations:** 1 Department of Applied Physics, Stanford University, Stanford, California 94305, USA; 2 Department of Materials Science and Engineering, Stanford University, Stanford, California 94305, USA; 3 Stanford Institute for Materials and Energy Sciences, SLAC National Accelerator Laboratory, 2575 Sand Hill Road Menlo Park, California, 94025, USA

## Abstract

Developing earth-abundant, active and stable electrocatalysts which operate in the same electrolyte for water splitting, including oxygen evolution reaction and hydrogen evolution reaction, is important for many renewable energy conversion processes. Here we demonstrate the improvement of catalytic activity when transition metal oxide (iron, cobalt, nickel oxides and their mixed oxides) nanoparticles (∼20 nm) are electrochemically transformed into ultra-small diameter (2–5 nm) nanoparticles through lithium-induced conversion reactions. Different from most traditional chemical syntheses, this method maintains excellent electrical interconnection among nanoparticles and results in large surface areas and many catalytically active sites. We demonstrate that lithium-induced ultra-small NiFeO_*x*_ nanoparticles are active bifunctional catalysts exhibiting high activity and stability for overall water splitting in base. We achieve 10 mA cm^−2^ water-splitting current at only 1.51 V for over 200 h without degradation in a two-electrode configuration and 1 M KOH, better than the combination of iridium and platinum as benchmark catalysts.

## Abstract (web summary)

There is intensive research underway in developing electrocatalysts for water splitting. Here, the authors present a lithium-induced conversion reaction method to develop an active and stable bifunctional catalyst for overall water splitting, outperforming the combination of noble metal catalysts.

## Graphical abstract

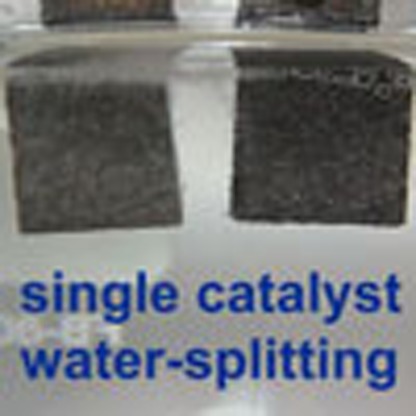

Electrochemical/photoelectrochemical water splitting is widely considered to be a critical step for efficient renewable energy production, storage and usage such as sustainable hydrogen production, rechargeable metal-air batteries and fuel cells^1^,^2^,^3^,^4^,^5^. Currently, the state-of-the-art catalysts to split water are IrO_2_ and Pt for oxygen evolution reaction (OER) and hydrogen evolution reaction (HER), respectively, with ∼1.5 V to reach 10 mA cm^−2^ current (for integrated solar water splitting)^1^,^6^. However, the price and scarcity of these noble metals present barriers for their scale-up deployment. A great deal of effort and progress have been made towards efficient OER and HER catalysts with earth-abundant materials, such as cobalt phosphate, perovskite oxides and transition metal oxides (TMOs)/layer-double-hydroxides for OER^7^,^8^,^9^,^10^,^11^,^12^,^13^, and transition metal dichalcogenides and nickel molybdenum alloy for HER^14^,^15^,^16^,^17^,^18^,^19^,^20^. However, pairing the two electrode reactions together in an integrated electrolyser for practical use is difficult due to the mismatch of pH ranges in which these catalysts are stable and remain the most active. In addition, producing different catalysts for OER and HER requires different equipment and processes, which could increase the cost. Therefore, developing a bifunctional electrocatalyst with high activity towards both OER and HER in the same electrolyte becomes important yet challenging. A recent work demonstrated an impressive water photolysis efficiency of 12.3% by using efficient NiFe layer double hydroxide bifunctional catalyst^21^. It was shown that 10 mA cm^−2^ overall water-splitting current was achieved in 1 M NaOH solution at ∼1.7 V (iR uncorrected) with a 470-mV overpotential from the equilibrium. Despite this exciting progress, new bifunctional materials with low overpotential and long-term stability are still needed. Here we demonstrate a novel bifunctional catalyst of lithium-induced ultra-small NiFeO_*x*_ nanoparticles (NPs), with a remarkable performance of only 1.51 V (280 mV overpotential) to achieve 10 mA cm^−2^ current in 1 M KOH solution for long-term operation.

We choose TMOs as candidates to develop bifunctional catalysts due to their good stability within a wide range of electrochemical window in base^13^,^22^,^23^. They have been shown as good catalysts for either OER or HER but there has not been an example that a single TMO can be an efficient catalyst for both reactions^13^,^22^,^24^. Previously our group has developed lithium-ion intercalation and extraction methods in battery cells to tune layered material catalysts, such as MoS_2_ and LiCoO_2_, and showed significant enhancement of catalytic activity for HER and OER, respectively^25^,^26^. Our hypothesis here is that the electrochemical lithium reaction method can tune the material properties of certain TMO catalysts to become highly active in both OER and HER for overall water splitting.

In this work, we explore a conversion reaction mechanism between Li and TMOs to improve the catalytic behaviour. Tarascon's work on lithium-ion batteries^27^ shows that, conversion reaction (MO+2 Li^+^+2 e^−^⇌M+Li_2_O) takes place by breaking the M–O bonds and forming M–M and Li–O bonds, which is different from the lithium interaction or extraction mechanism employed in our previous studies^25^,^26^. Conversion reaction can cause dramatic change in the MO materials (Fig. 1). Once lithium is extracted to reform MO, the initial MO particles would transform into much smaller ones with few nanometres in diameter (Fig. 1)^27^. This morphological transformation opens up opportunities to increase the surface area of TMOs tremendously. With the limited number of lithium galvanostatic cycles, these small particles can be maintained interconnected (Fig. 1c,d). We assume that the ultra-small, interconnected TMO NPs present an ideal structure for highly active and stable electro water splitting because they create a great number of grain boundaries as active centres, expose additional catalytically active sites and strongly interact with each other during the delithiation reaction process which helps to maintain good mechanical and electrical contacts. However, large number of battery cycles may break off the particles, resulting in the loss of connection and form a thick solid electrolyte interface covering the surface^27^, which induce negative effects on the catalytic performance of TMOs (Fig. 1e). Therefore, we will need to limit the number of battery cycles to select the most active catalyst. Herein, we demonstrate the general efficacy of lithium galvanostatic cycling in improving OER catalytic activities of TMOs (M=Fe, Co, Ni, and their mixture). High-performance OER catalyst is then selected to show the enhanced HER activity. With two half reactions greatly improved by the galvanostatic cycling method, efficient and stable overall water splitting by the bifunctional catalyst is presented.

## Results

### TMO NPs on carbon nanofibres for characterizations

**Figure 1:**
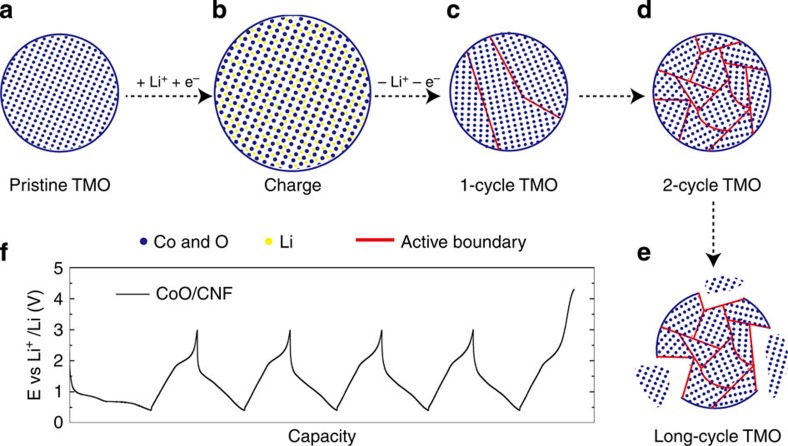
Schematic of TMO morphology evolution under galvanostatic cycles. (**a**–**e**) TMO particles gradually change from single crystalline to ultra-small interconnected crystalline NPs. Long-term battery cycling may result in the break-up of the particle. (**f**) The galvanostatic cycling profile of CoO/CNF galvanostatic cycling.

**Figure 2:**
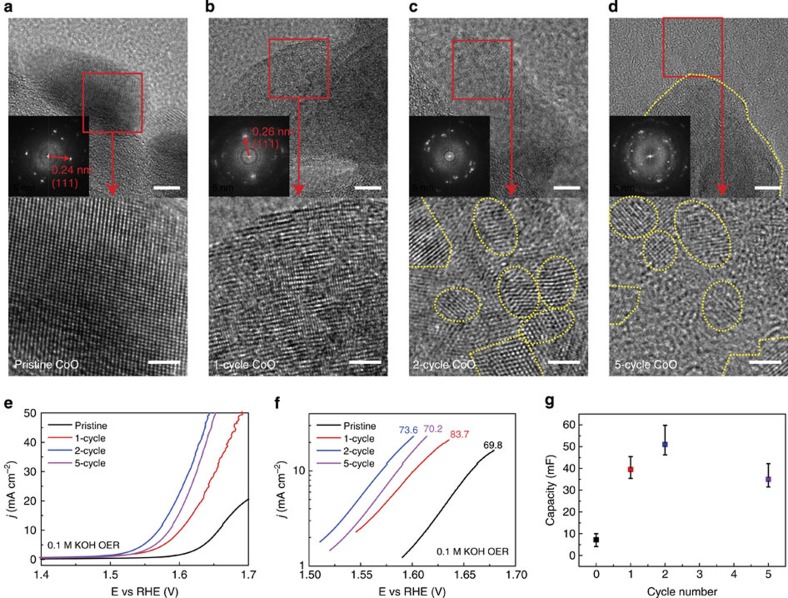
TEM images and OER activities of battery-cycled CoO/CNF. (**a**) TEM image of pristine CoO/CNF. The lattice structure and the FFT pattern indicate the single-crystalline nature of the pristine particle. (**b**) With a blurred lattice orientation still visible, TEM image of 1-cycle CoO/CNF exhibits defects, lattice distortions and expanded (111) spacing. (**c**) TEM image of 2-cycle CoO/CNF shows the ultra-small, interconnected NPs. The sizes are ∼2–5 nm. (**d**) TEM image of 5-cycle CoO/CNF shows similar domain size to the 2-cycle one. The yellow dash line in the upper image represents the boundary of the whole particle. The zoom-in image indicates the detachment of the ultra-small NPs from the mother particle. Scale bars in **a**–**d**, upper, 5 nm; lower, 2 nm. (**e**) OER catalytic activities of CoO/CNF on CFP in 0.1 M KOH under different galvanostatic cycles. The polarization scan rate is 5 mV s^−1^. Two-cycle CoO/CNF gives the best performance. (**f**) The Tafel plots of OER polarization curves. (**g**) Electrochemical double layer capacitance of CoO/CNF under different cycles. The error bars include three identical samples tested for each cycle number.

We first grow CoO NPs on carbon nanofibres (CNFs) to study the morphology evolutions and the corresponding improvements in OER activities under different galvanostatic cycle numbers (see Methods). The pristine CoO NPs are ∼20 nm in diameter and uniformly distributed on CNFs (Supplementary Fig. 1, we denote this sample as pristine CoO/CNF). Transmission electron microscopy (TEM) and the corresponding fast Fourier transform (FFT) images suggest the monocrystalline nature of pristine CoO NPs (Fig. 2a). The spacing of (111) atomic planes is measured to be 0.24 nm, consistent with previous studies^28^. The CoO/CNF was then assembled in a lithium-ion battery pouch cell for galvanostatic lithiation (charge) and delithiation (discharge) processes (Fig. 1f, see Methods)^27^. Small charge/discharge current (compared with regular battery cycling) was selected for thorough reaction (see Methods), which also helps to maximally maintain the integration of the particles for long-term stability. The morphology of CoO begins to change after one cycle of the charge/discharge process (we denote the cycled samples as 1-cycle, 2-cycle and 5-cycle CoO/CNF). While the whole lattices are still visible, the fringes become curvy and loose compared with pristine CoO (Fig. 2b). Defects are created during the cycling process, as suggested by the blurred areas present in the zoomed-in TEM image. The average (111) spacing of 1-cycle CoO is ∼0.26 nm, slightly expanded from the pristine 0.24 nm. This lattice expansion and distortion in the first cycle lower the energy barrier for a small lattice domain to change orientation, preparing for the large particle to be further transformed into smaller particles in the following cycles. The TEM images of 2-cycle CoO/CNF show that the monocrystalline CoO particle is converted into interconnected crystalline NPs, with ultra-small sizes ∼2 nm (Fig. 2c). The FFT image with significantly more diffraction spot patterns than pristine CoO also suggest that many lattice orientations are present in this single CoO particle. The ultra-small NPs create boundaries, defects and dislocations, which are considered to be active sites of electrocatalysis^29^. Two neighbouring NPs merge together at the boundary without any visible gaps present, suggesting that they are strongly interconnected with each other that ensures good electrical and mechanical contact for efficient and stable catalysis. Similar structures are also observed in NiO, FeO and NiFeO_*x*_ NPs (Supplementary Fig. 2). As indicated by the TEM images of 5-cycle CoO/CNF (Fig. 2d and Supplementary Fig. 3), further cycles do not significantly reduce the sizes of the interconnected NPs or even convert them into amorphous, suggesting that ultra-small NPs have reached the minimum domain sizes under the specific cycling condition. In areas away from the integrated particle, we observe that several ultra-small CoO crystals are detached, which indicates that more cycling number harms the integration of the whole particle and may also loosen the contacts between the interconnected NPs. X-ray diffraction spectroscopy of pristine CoO has three distinguished peaks, which however disappear in all of the battery-cycled samples (Supplementary Fig. 4), indicating that the sizes of the interconnected NPs are under the X-ray coherence length^27^. Raman spectra of pristine and 2-cycle CoO/CNF confirm that the phase of CoO is not changed after the battery cycling process (Supplementary Fig. 5)^28^.

To examine the electrochemical OER catalytic activities, pristine CoO/CNF were drop casted onto commercial carbon fibre paper (CFP) substrates, followed by 1, 2 and 5 galvanostatic cycles, respectively (Supplementary Fig. 6, see Methods). The as-prepared catalysts were tested in 0.1 M KOH solution. All of the potentials are referred to reversible hydrogen electrode (RHE) and have been iR corrected unless noted (see Methods). Pristine CoO/CNF shows a sluggish OER process with an onset potential around 1.59 V and a Tafel slope of 69.8 mV per decade (Fig. 2e). The activity of 1-cycle CoO/CNF is significantly improved, achieving a reduced onset potential to ∼1.55 V while exhibiting a slightly increased Tafel slope of 83.7 mV per decade. The increased surface area, atomic defects and distortions created during the first cycle in Fig. 2b are considered to contribute to the improved catalytic activity. The OER performance is continuously improved after two galvanostatic cycles, as additional surface areas and active sites are introduced by those ultra-small interconnected NPs (Fig. 2c,e). While the Tafel slope (73.6 mV per decade) of 2-cycle CoO/CNF is not changed much, the onset potential is further lowered to ∼1.51 V, significantly improving the OER activity which reaches 10 mA cm^−2^ anodic current at ∼1.57 V. Five-cycle CoO/CNF shows a degraded OER performance compared with the 2-cycle sample, consistent with the analysis of the TEM image (Fig. 2d) that some of the ultra-small NPs are detached from and lose electrical contact with the mother particle. The electrochemical double layer capacities of the catalysts, which represent the active surface areas, are obtained by applying cyclic voltammograms at a series of scanning rates (Fig. 2g and Supplementary Figs 7 and 8). The trend of the capacity versus the cycle number agrees well with that of the OER activity, where 2-cycle CoO/CNF exhibits the largest capacity. Therefore, we conclude that two galvanostatic cycles is an optimized condition for improving the catalytic performance of as-synthesized TMO NPs. While the conversion from monocrystalline particle to polycrystalline NPs helps to significantly increase the active sites and surface areas, whether those ultra-small crystalline NPs become amorphous under the OER conditions is worth to be further examined. The TEM image of 2-cycle CoO/CNF after OER catalysis is shown in Supplementary Fig. 9, in which the structures and sizes of interconnected crystalline NPs are well maintained and no sign of amorphization process is observed. No Li signal is observed in 2-cycle CoO/CNF by electron energy loss spectroscopy as shown in Supplementary Fig. 10, indicating that the concentration of residual Li is lower than the electron energy loss spectroscopy detection limit. In addition, the molar ratio of Li to Co in 2-cycle CoO/CNF is determined to be 1:23.4 by inductive coupled plasma mass spectroscopy, suggesting the negligible amount of residual Li after the cycling process. To shed light on how Li doping influences the catalytic activities, we doped CoO/CNF with Li by charging the electrode to 1 V versus Li^+^/Li (right above the conversion reaction plateau, Li to Co ratio was determined to be 1:7 by inductive coupled plasma mass spectroscopy). The OER performance shows a slightly decay compared with pristine CoO/CNF in Supplementary Fig. 11, indicating that Li doping does not contribute to the improvement in OER performance. Combined with the analysis of the great contributions from the increased surface areas as well as capacitances, we therefore conclude that the very small amount of residual Li does not play a role in improving the OER catalysis. We also rule out the possibility of background contributions by performing battery cycling on bare CNF in Supplementary Fig. 12.

### TMO NPs synthesized on CFP for high-performance catalysis

**Figure 3:**
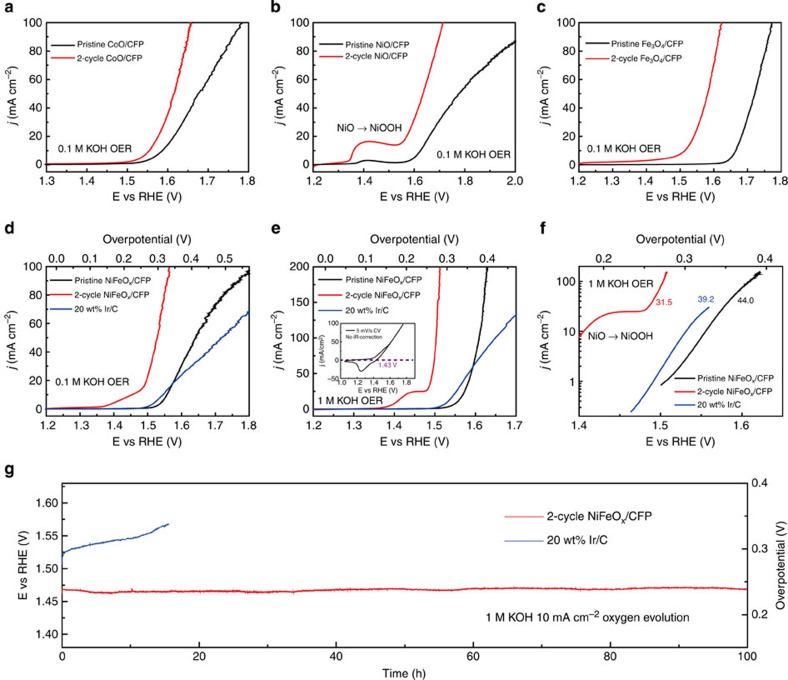
OER activities and stability of pristine and 2-cycle TMO/CFP catalysts. (**a**–**d**) The general efficacy of galvanostatic cycling in improving the OER activities of Co, Ni, Fe and NiFe oxides in 0.1 M KOH. Two-cycle NiFeO_*x*_/CFP exhibits better performance than the Ir/C benchmark. (**e**,**f**) The OER polarization curves and the corresponding Tafel plots of pristine and 2-cycle NiFeO_*x*_/CFP in 1 M KOH. The polarization scanned from positive potential to negative in the inset indicates the onset potential of 2-cycle NiFeO_*x*_/CFP at ∼1.43 V. The Tafel slope of 2-cycle NiFeO_*x*_/CFP is 31.5 mV per decade, better than the Ir/C benchmark. (**g**) Two-cycle NiFeO_*x*_/CFP exhibits an excellent OER stability, achieving 10 mA cm^−2^ anodic current at only 1.46 V versus RHE for over 100 h without degradation. This is better than the Ir/C benchmark.

To avoid the long-term stability and large current bubble-releasing issues of TMO NPs on CNF (due to the use of binder and the hydrophobic nature of carbon respectively), we directly synthesize TMO catalysts on CFP substrates including CoO/CFP, NiO/CFP, Fe_3_O_4_/CFP and the mixed oxide of NiFeO_*x*_/CFP (Supplementary Figs 13 to 16, see Methods). The mass loadings of the catalysts are ∼1.6 mg cm^−2^ and the Ir and Pt benchmarks are 0.5 mg cm^−2^ (Supplementary Fig. 17, see Methods). Galvanostatic cycling shows its general efficacy in improving all of the TMOs from their pristine counterparts, with significantly reduced onset potentials as well as overpotentials to achieve 20 mA cm^−2^ OER current (Fig. 3a–d and Supplementary Figs 18–20). It is interesting to notice that 2-cycle NiO/CFP shows a significantly increased NiO to NiOOH oxidation peak, again confirming the impressively increased surface areas and active sites, which suggests the potential applications of the galvanostatic cycling method in supercapacitors^30^. The best OER performance comes from 2-cycle NiFeO_*x*_/CFP (Fig. 3d and Supplementary Fig. 18). In 0.1 M KOH, 20 wt% Ir/C reaches 10 and 20 mA cm^−2^ at ∼1.53 and 1.58 V, respectively. As a comparison, the OER activity of 2-cycle NiFeO_*x*_/CFP outperforms this noble metal, with only 1.48 (*η*_OER 10 mA_=250 mV) and 1.50 V (*η*_OER 20 mA_=270 mV) to achieve the corresponding currents (Supplementary Figs. 18e,f). This highly efficient catalyst exhibits even better OER performance as pH increases to 14 (1 M KOH; Fig. 3e). To avoid the overlap of the NiO to NiOOH oxidation peak with the OER onset currents, we scanned the voltage from the positive to the negative direction (the inset of Fig. 3e) and determine the onset potential of 2-cycle NiFeO_*x*_/CFP in 1 M KOH to be ∼1.43 V (*η*_OER onset_=200 mV), nearly 40 mV lower than Ir/C. The OER current of 2-cycle NiFeO_*x*_/CFP then ramps up quickly to 200 mA cm^−2^ at only 1.51 V. This high OER activity benefits from the small Tafel slope of 31.5 mV per decade which does not show the curve bending as observed in pristine NiFeO_*x*_/CFP and Ir/C, suggesting the improved kinetic and bubble-releasing processes by galvanostatic cycling (Fig. 3f). To avoid the oxidation peak and therefore obtain a larger range of current for NiFeO_*x*_/CFP Tafel slope analysis, we reversely swept the *I–V* curve as shown in Supplementary Fig. 21 and calculated the Tafel slope to be 34.2 mV per decade, very close to the forward sweeping result. It is worth noting that the voltage sweeping rate in all of the tests is 5 mV s^−1^, which is slow enough to reach the steady state for accurate analysis of Tafel slopes (Supplementary Fig. 22). The reverse scanning method also helps to reveal an interesting conclusion that in more concentrated KOH solution the oxidation process can go deeper on the surface of the NiFeO_x_ catalyst (Supplementary Fig. 23). Very small oxidation peaks of CoO and Fe_3_O_4_ were also observed in Supplementary Fig. 24. Stability of the battery-cycled TMO is of our concern that whether these ultra-small interconnected NPs can tolerate the violent condition of gas evolution. An impressive OER stability of 2-cycle NiFeO_*x*_/CFP is shown in Fig. 3g, with 10 mA cm^−2^ anodic current at ∼1.46 V (*η*_OER 10 mA_=230 mV) for over 100 h without degradation. The high activity and long-term stability confirm the strong interactions between those ultra-small, interconnected NPs, outperform the OER catalysts reported so far, and consequently makes this material attractive for practical use in the future.

**Figure 4:**
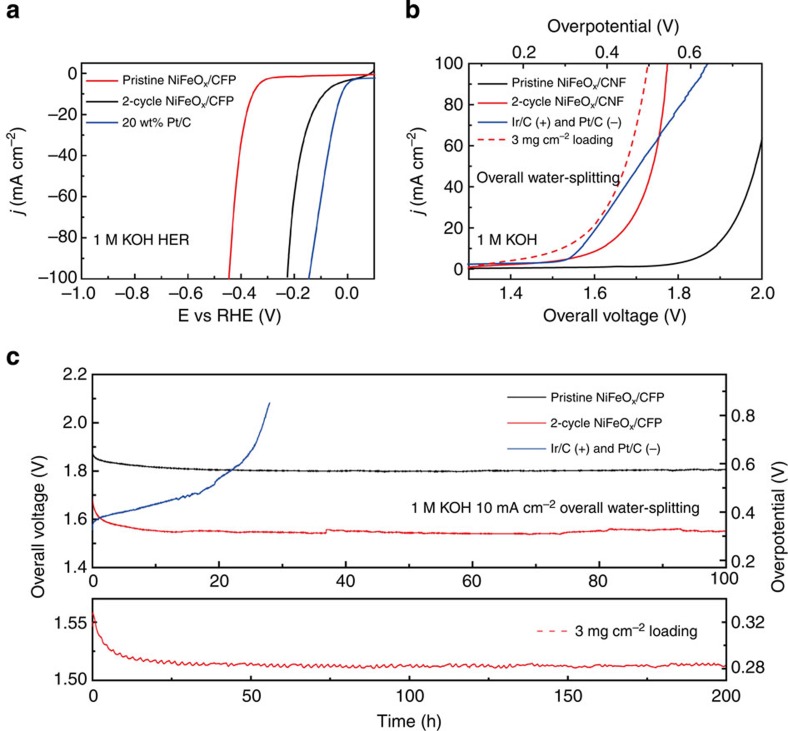
Bifunctional 2-cycle NiFeO_*x*_/CFP for two-electrode water splitting. (**a**) The HER activity of 2-cycle NiFeO_*x*_/CFP is significantly improved from its pristine counterpart and close to the Pt/C benchmark. (**b**) Two-cycle NiFeO_*x*_/CFP as HER and OER bifunctional catalyst in 1 M KOH for overall water splitting. Ir/C and Pt/C as OER and HER benchmarks are tested side by side. With the mass loading increased from 1.6 to 3 mg cm^−2^ (the dash line), the water-splitting activity of 2-cycle NiFeO_*x*_/CFP outperforms the benchmark combination. (**c**) Long-term stability of 2-cycle NiFeO_*x*_/CFP bifunctional catalyst. The voltage to achieve 10 mA cm^−2^ electrolysis current shows an activation process, followed by a stable 1.55 V for 100-h continuous operation. As a sharp contrast, Ir and Pt combination shows an efficient starting voltage but followed by a fast decay. By increasing the mass loading to 3 mg cm^−2^, 2-cycle NiFeO_*x*_/CFP further lowers the voltage to 1.51 V to achieve 10 mA cm^−2^ current for over 200 h without decay.

Efficient HER catalysts in alkaline solutions such as transition metals and their alloys have been well investigated^15^,^16^,^17^, but the HER activities of TMOs are rarely developed^22^, which limits the study of high-performance bifunctional OER and HER catalysts for overall water splitting. The HER activity of 2-cycle NiFeO_*x*_/CFP as an efficient OER catalyst is also tested in 1 M KOH, which shows a small onset potential of −40 mV, significantly improved from its pristine counterpart with a large onset of −310 mV (Fig. 4a). The Tafel slope increases from 84.6 to 150 mV per decade after the battery cycling process, which may be related to a change of the reaction pathway or a mass transport limit (Supplementary Fig. 25)^31^,^32^. A small overpotential of −88 mV is required for 2-cycle NiFeO_*x*_/CFP to reach −10 mA cm^−2^ cathodic current, which is not far from the Pt benchmark of −23 mV. Together with the other half reaction of OER, the galvanostatic cycling method creates an attractive bifunctional NiFeO_*x*_/CFP water-splitting catalyst to compete with the combination of Pt and Ir benchmarks. The overall water-splitting polarization of 2-cycle NiFeO_*x*_/CFP bifunctional catalyst exhibits a slightly larger onset voltage than the benchmark combination, but quickly catches up with them due to the facile kinetic and bubble-releasing processes (Fig. 4b). In addition, the sizes of O_2_ and H_2_ bubbles observed on 2-cycle NiFeO_*x*_/CFP electrodes under 200 mA cm^−2^ are distinctively smaller than those on the benchmark electrodes, indicating the great capability for large current operations (Supplementary Movie 1). The long-term stability testing further illustrates the advantages of 2-cycle NiFeO_x_/CFP over those noble metals (Fig. 4c). With a slightly higher starting voltage to achieve 10 mA cm^−2^ of constant water-splitting current, 2-cycle NiFeO_*x*_/CFP exhibits a gradually increased catalytic activity and surpasses the benchmark combination after 1-h operation. Gas chromatography measurements of 2-cycle NiFeO_*x*_/CFP water electrolysis confirm a high faradic efficiency of O_2_ and H_2_, calibrated by the benchmark electrodes (Supplementary Fig. 26). During the long-term stability testing, it is possible for the oxidation process (MO to MOOH) to get deeper at a very slow rate, gradually reaching to a limit. This may help to create additional active sites and refresh the boundaries of the interconnected particles, which slightly increases the activity. The gas evolution may also help to remove surface residues from the battery cycling, which contribute to the activation process observed^22^. The voltage stabilizes at ∼1.55 V (*η*_overall 10 mA_=320 mV) for 100-h continuous operation, in a sharp contrast to the benchmark combination. In addition, the water-splitting performance of our catalyst can be further improved simply by increasing the mass loading to 3 mg cm^−2^ (Fig. 4b,c, see Methods). The high-mass catalyst further brings the voltage down to 1.51 V (*η*_overall 10 mA_=280 mV) to achieve 10 mA cm^−2^ current, with remarkable stability of over 200 h with no sign of decay. Overall water splitting in neutral electrolyte is also tested in Supplementary Fig. 27, which however shows much lower activity compared with that in the alkaline solution.

## Discussion

By improving both OER and HER activities, the galvanostatic cycling method successfully elevates the efficiency of water-splitting electrolyser at 10 mA cm^−2^ current to 81.5% using only one material, making good preparations for the scale-up of water photolysis/electrolysis with high efficiency and low cost^21^. Synthesizing catalysts on conducting substrates can maximally reduce the use of carbon additives and also get rid of polymer binders, which enables high-current operations (circumvent bubble-releasing problems introduced by the hydrophobic nature of carbon) and also performs superior stabilities. In addition, the successful demonstration of the Li conversion reaction method in improving water-splitting catalysis may help to inspire the improvements of other important TMOs applications including oxygen reduction reactions, supercapacitors, carbon dioxide reductions and so on.

## Methods

### CNF synthesis

Polyacrylonitrile (0.5 g, PAN, *M*_w_=150,000, Sigma-Aldrich) and 0.5 g polypyrrolidone (PVP, *M*_w_=1,300,000, Sigma-Aldrich) were dissolved in 10 ml of dimethylformamide under 80 °C with constant stirring. The solution was electrospun using a conventional electrospinning set-up with the following parameters—15 kV of static electric voltage, 18 cm of air gap distance, 3 ml PVP and PAN solution and 0.5 ml h^−1^ flow rate. A carbon fibre paper substrate (8 × 8 cm) was used as the collection substrate. The electronspun polymer nanofibres on the carbon fibre paper was then heated up to 280 °C in 30 min in the box furnace, and kept under the temperature for 1.5 h to oxidize the polymers. After the oxidization process, the nanofibres were self-detached from the carbon paper resulting in the freestanding film. Those nanofibres were carbonized under argon atmosphere at 900 °C for 2 h to become a CNF matrix.

### CoO/CNF synthesis

The solution of cobalt nitrate were first prepared by dissolving 25 wt% Co(NO_3_)_2_·6H_2_O (Sigma-Aldrich) and 1 wt% PVP (M_w_=360,000, Sigma-Aldrich) into 56 wt% deionized water. Specifically, 1.25 g of Co(NO_3_)_2_·6H_2_O and 0.05 g of PVP were dissolved into 3.7 ml of deionized water. O_2_ plasma-treated CNF matrix was then dipped into the solution and dried in the vacuum for overnight. The Co(NO_3_)_2_/CNF was then heated up to 500 °C in 1 h under 1 a.t.m. Ar atmosphere with a slow flow rate of 10 s.c.c.m. in a tube furnace and kept there for 1.5 h, where the Co(NO_3_)_2_ was decomposed into CoO NPs. The mass ratio of CoO to CNF is 0.24.

### TMO/CFP synthesis

TMO NPs are directly synthesized on CFP electrode (AvCarb MGL190, FuelCellStore) by the same dip-coating method mentioned above. Specifically, 4 g of transition metal nitrite (40 wt%) and 0.4 g of PVP (4 wt%) were dissolved into 5.6 ml deionized water. The mixture of Ni(NO_3_)_2_·6H_2_O and Fe(NO_3_)_3_·9H_2_O was based on the molar ratio of 3:1. The thermal decomposition process is the same with CoO/CNF synthesis. The high temperature during the synthesis helps to create strong bonds between the catalysts and substrates, which can greatly benefit their stabilities. The mass loading of the TMOs on CFP is ∼1.6 mg cm^−2^. Large mass loading of 3 mg cm^−2^ is obtained by using the CFP substrate with larger surface areas (AvCarb MGL370, FuelCellStore).

### OER electrode preparation

CoO/CNF was first put into a stainless steel vial for 20 min ball milling (5100 Mixer/Mill, SPEX SamplePrep LLC). These small pieces with nafion (Nafion 117 solution, Sigma-Aldrich) were then dispersed into ethanol with a concentration of 5 mg ml^−1^. The mass ratio of CoO/CNF to nafion is 10:1. The solution was then drop onto CFP electrode with a mass loading of 0.6 mg cm^−2^ (based on the CoO/CNF). The preparations of Ir/C (20 wt% Ir on Vulcan XC-72, Premetek Co.) and Pt/C (20 wt% Pt on Vulcan, FuelCellStore) inks are the same with that of CoO/CNF. The mass loading of Ir and Pt on CFP is 0.5 mg cm^−2^. More loading may result in severe bubble-releasing problems due to the high concentration of carbon (Supplementary Fig. 17).

### Galvanostatic cycling

The as-grown CoO on CNF matrix was made into a pouch cell battery with a piece of Li metal and 1.0 M LiPF_6_ in 1:1 w/w ethylene carbonate/diethyl carbonate (EMD Chemicals) as electrolyte. The galvanostatic cycling current is set at 173 mA g^−1^ and cycle between 0.4 and 3 V versus Li^+^/Li. The cutoff voltage of the last discharging step is 4.3 V for thoroughly delithiation. The galvanostatic cycled CoO on CNF matrix is then washed by ethanol for SEM, X-ray diffraction and Raman and sonicated into small pieces for TEM characterizations. CoO/CNF on CFP is cycled at 0.1 mA cm^−2^ current and TMO/CFP catalysts electrodes are cycled at 62.5 mA g^−1^ current.

### Electrochemical characterizations

All of the electrochemical tests are performed under 1 a.t.m. in air and room temperature 25 °C. OER, HER and electrochemical double layer capacitance are tested in a three-electrode set-up and overall water splitting is performed in a two-electrode system. Saturated calomel electrode is selected as the reference electrode with a potential of 0.99 V versus RHE in 0.1 M KOH, 1.049 V versus RHE in 1 M KOH, and 1.131 V versus RHE in 6 M KOH calibrated by purging pure H_2_ gas on the Pt wire. We use Pt wire and Ni foam as counter electrodes for OER and HER tests, respectively. In the two-electrode full cell, one 2-cycle NiFeO_*x*_/CFP (or pristine NiFeO_*x*_/CFP) electrode acts as the positive electrode for OER and the other 2-cycle NiFeO_*x*_/CFP (or pristine NiFeO_*x*_/CFP) electrode act as the negative electrode for HER. For the benchmark control, Ir/C acts as positive electrode and Pt/C as negative electrode. The impedance spectra of OER in three-electrode system are tested under 1.5 V versus RHE in 0.1 M KOH and 1.45 V versus RHE in 1 M KOH with an example of NiFeO_*x*_/CFP in Supplementary Fig. 20. The HER impedance is tested under −0.05 V versus RHE. The impedance of the two-electrode full cell is tested under 1.5 V voltage. All of the potentials and voltages are iR corrected unless noted. The two-electrode full cell stability testing was performed in a 100-ml lab bottle with two electrodes located around 3–5 cm away from each other to prevent the crossover of the gas products. The bottle was open to the air during the testing to release the produced H_2_ and O_2_. All of the polarization curves were obtained at the scanning rate of 5 mV s^−1^.

## Additional information

**How to cite this article:** Wang, H. *et al.* Bifunctional non-noble metal oxide nanoparticle electrocatalysts through lithium-induced conversion for overall water splitting. *Nat. Commun.* 6:7261 doi: 10.1038/ncomms8261 (2015).

## Supplementary Material

Supplementary Figures and Supplementary References Supplementary Figures 1-27 and Supplementary References.

Supplementary Movie 1 Bubble evolution of water splitting
