# Supplementary material for: Bifunctional non-noble metal oxide nanoparticle electrocatalysts through lithium-induced conversion for overall water splitting
Source: Nat Commun. 2015 Jun 23;6:7261. doi: 10.1038/ncomms8261 (PMC4557299; doi:10.1038/ncomms8261)
Supplement: Supplementary Figures and Supplementary References — Supplementary Figures 1-27 and Supplementary References. [file ncomms8261-s1.pdf]

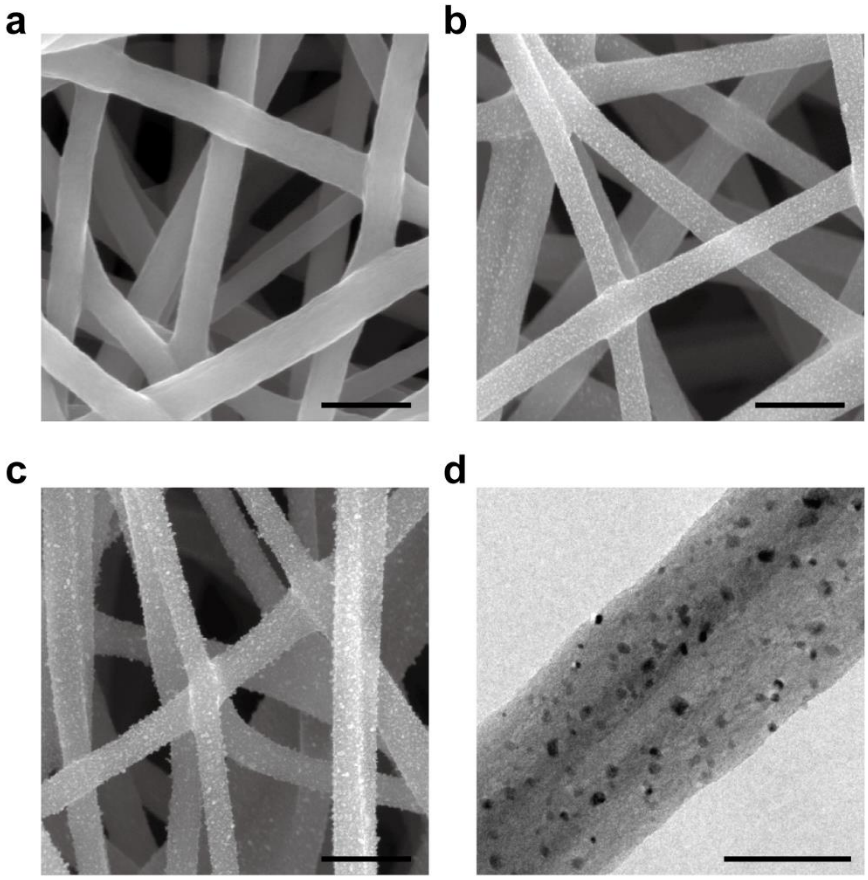

Supplementary Figure 1. SEM and TEM images of CoO/CNF before and after galvanostatic cycles. (**a**) SEM image of CNF. (**b**) SEM image of CoO NPs uniformly distributed on CNF. (**c**) SEM image of 2-cycle CoO/CNF. (**d**) TEM image of pristine CoO/CNF suggesting the uniform sizes and distributions of the NPs. Scale bars: (**a**), (**b**), (**c**), 500 nm; (**d**), 200 nm.

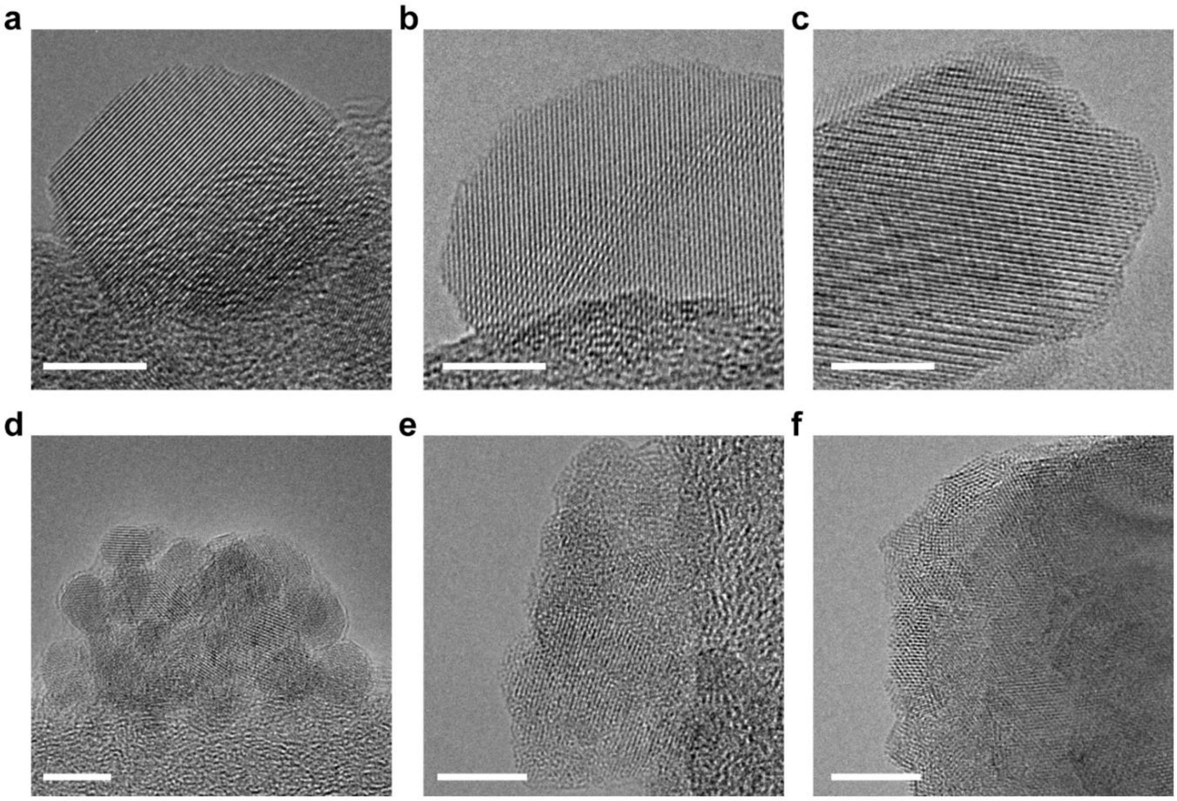

Supplementary Figure 2. TEM images of TMOs before and after 2 battery cycles. (**a**), (**d**) TEM images of pristine and 2-cycle NiO/CNF respectively. (**b**), (**e**) TEM images of pristine and 2-cycle Fe_3O_4/CNF respectively. (**c**), (**f**) TEM images of pristine and 2-cycle $NiFeO_x$/CNF respectively. All of the transition metal oxides including CoO/CNF in Fig. 2 shows the transformation from monocrystalline particles into ultra-small interconnected crystalline nanoparticles. Scale bars: 5 nm.

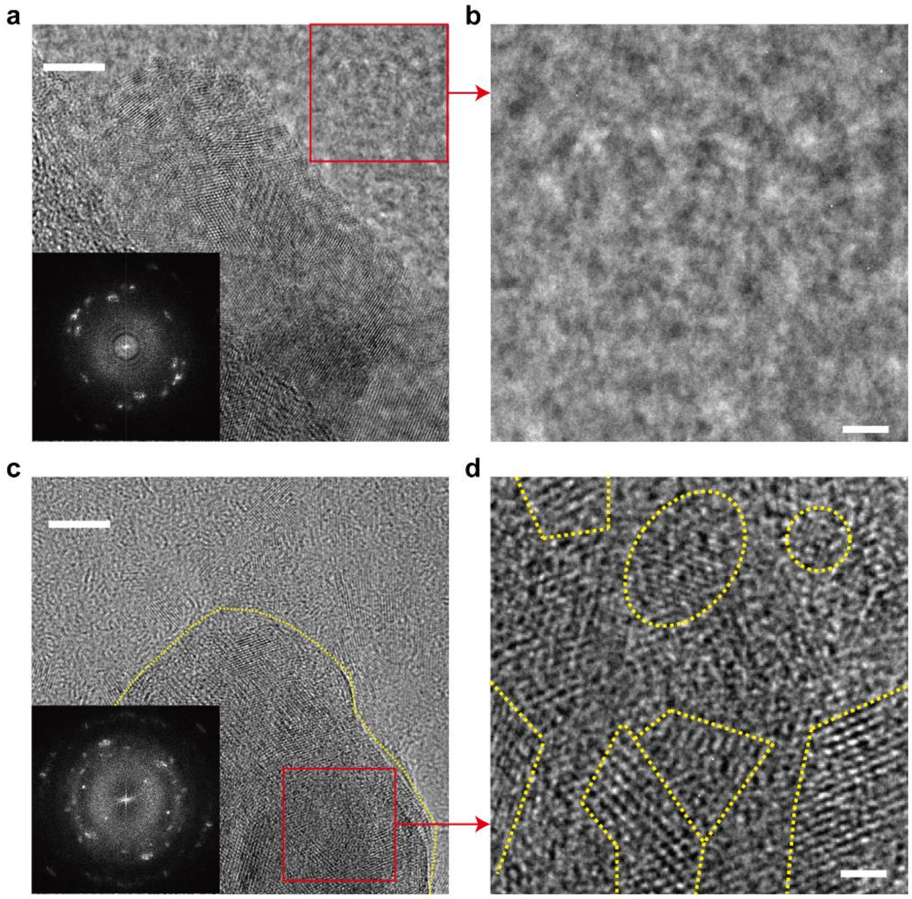

Supplementary Figure 3. TEM images of 2-cycle and 5-cycle CoO/CNF. (**a**), (**b**) The zoomed-in area away from the 2-cycle CoO particle is featureless, indicating that the ultra-small NPs are strongly interconnected. (**c**), (**d**) The zoomed-in area of 5-cycle CoO particle suggests the domain size of the ultra-small NPs is similar to 2-cycle CoO/CNF sample. Scale bars: (**a**), (**c**), 5 nm; (**b**), (**d**), 1 nm.

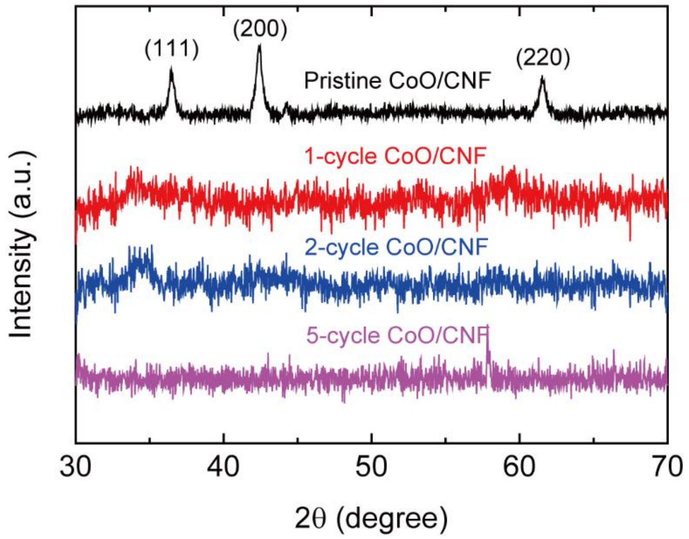

Supplementary Figure 4. XRD spectra of pristine, 1-cycle, 2-cycle, and 5-cycle CoO/CNF. Pristine CoO/CNF shows three distinguished peaks representing (111), (200), and (220) surfaces. The XRD spectra of other samples after galvanostatic cycling are featureless, indicating that the sizes of the NPs are below the coherence length of the X-ray.

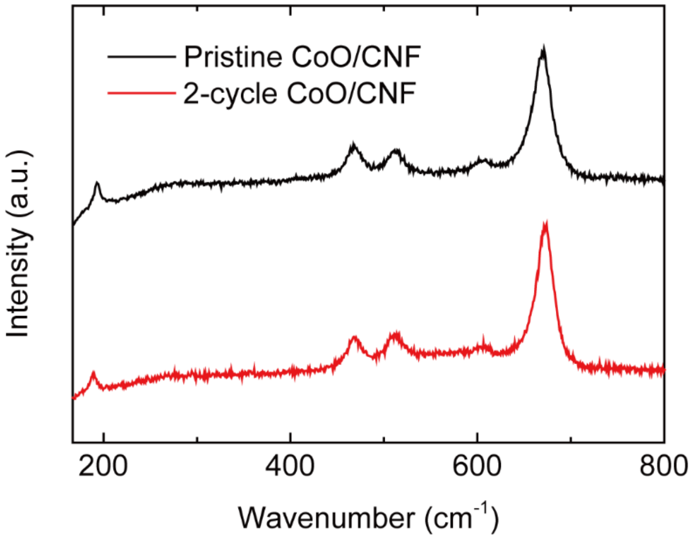

Supplementary Figure 5. Raman spectra of pristine and 2-cycle CoO/CNF[1]. The distinguished peaks before and after galvanostatic cycling are similar, indicating that the chemical composition and phase of CoO is not changed after the cycling process. In addtion, no broadening of the peaks is observed even though the particles sizes are significantly reduced. This might be related to the specific structure after the treatment of battery cycling. Different from those separated particles, the ultra-small nanoparticles are strongly connected with each other and form an integrated secondary particle (TEM images in Fig. 2). The grain boundary conditions of those ultra-small nanoparticles are therefore completely different from the case of separated particles. The strongly interacted boundaries may help to enhance the long distance translational periodicity, resulting in a similar Raman spectrum to the pristine one.

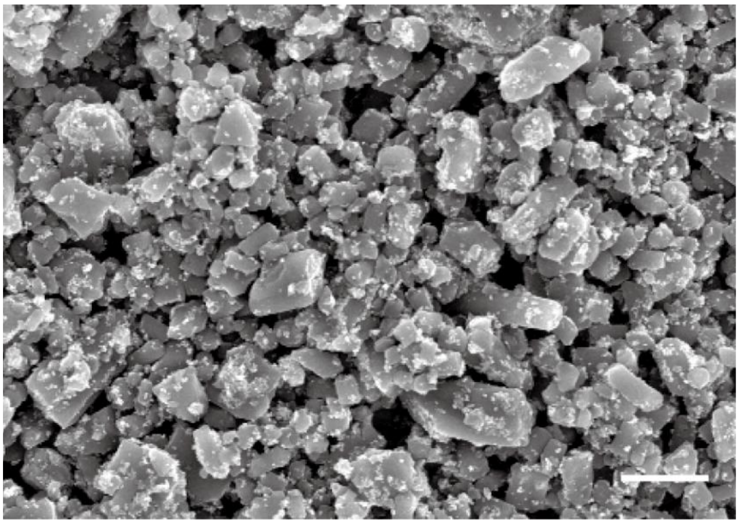

Supplementary Figure 6. SEM image of ball milled CoO/CNF particles with sizes ranging from 200 nm to ~ 1 μm. Scale bar: 1 um.

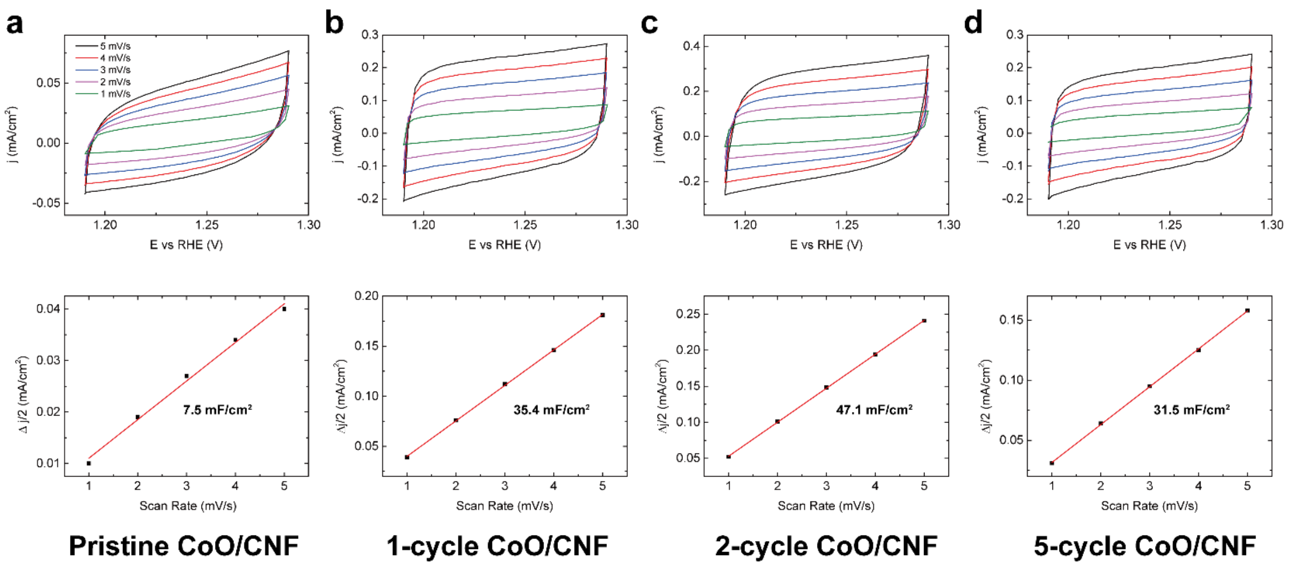

Supplementary Figure 7. ECDLC measurements of CoO/CNF samples. (**a**) ECDLC of pristine CoO/CNF with a capacitance of 7.5 mF/cm^2. (**b**) ECDLC of 1-cycle CoO/CNF with an increased capacitance of 35.4 mF/cm^2. (**c**) 2-cycle CoO/CNF shows the highest capacitance of 47.1 mF/cm^2. (**d**) The ECDLC of 5-cycle CoO/CNF slightly decreases from its 2-cycle counterpart, with a capacitance of 31. 5 mF/cm^2.

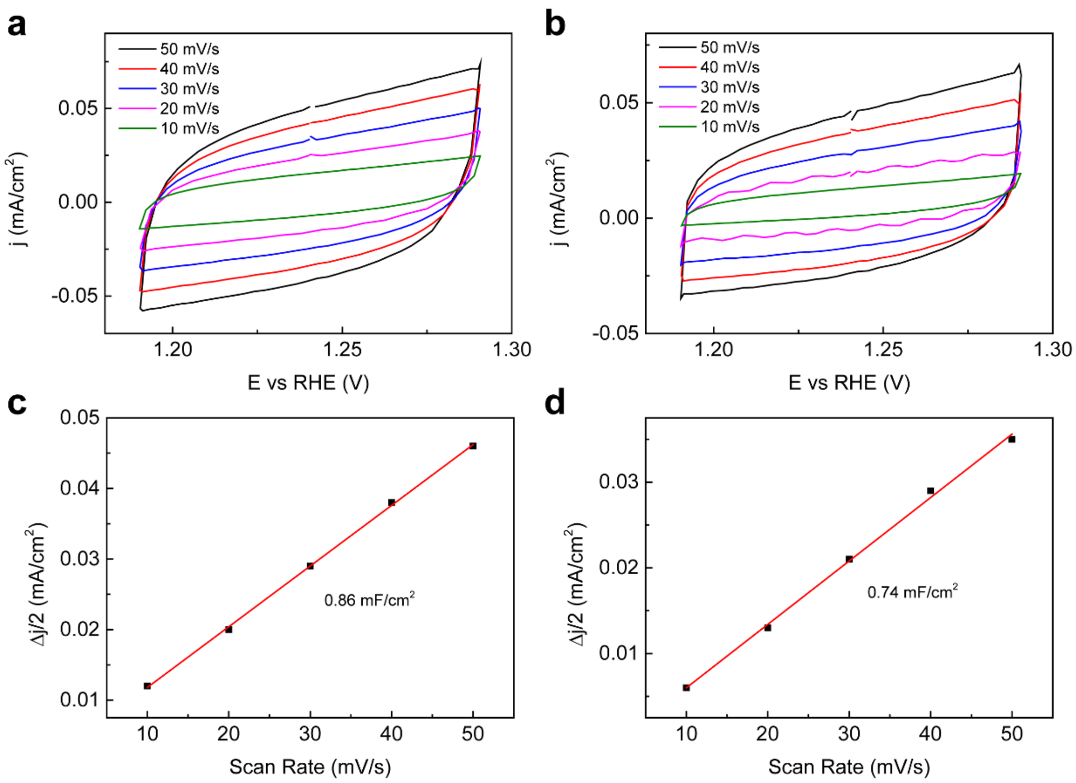

Supplementary Figure 8. ECDLC measurements of CNF backgrounds before and after 2 battery cycles. (**a**), (**c**) ECDLC of pristine CNF loaded on CFP with a capacitance of 0.86 mF/cm^2. (**b**), (**d**) ECDLC of 2-cycle CNF on CFP with roughly the same capacitance of 0.74 mF/cm^2. Both capacitances are negligible compared with the catalysts in Supplementary Fig. 7.

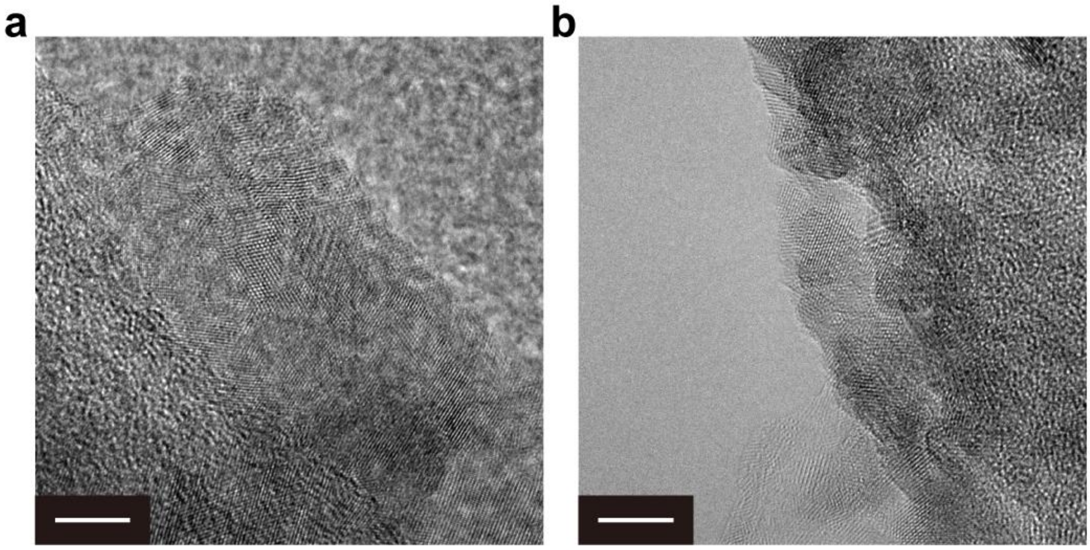

Supplementary Figure 9. TEM images of 2-cycle CoO/CNF before and after OER. (**a**) TEM image of 2-cycle CoO/CNF. (**b**) TEM image of 2-cycle CoO/CNF after oxygen evolution under a potential of 1.65 V vs RHE for 30 min. The structures of interconnected crystalline nanoparticles are well maintained, and their sizes are not changed much compared with the sample before OER. This indicates that it is less likely for the amorphization process to take place under the OER condition. Scale bars: 5 nm.

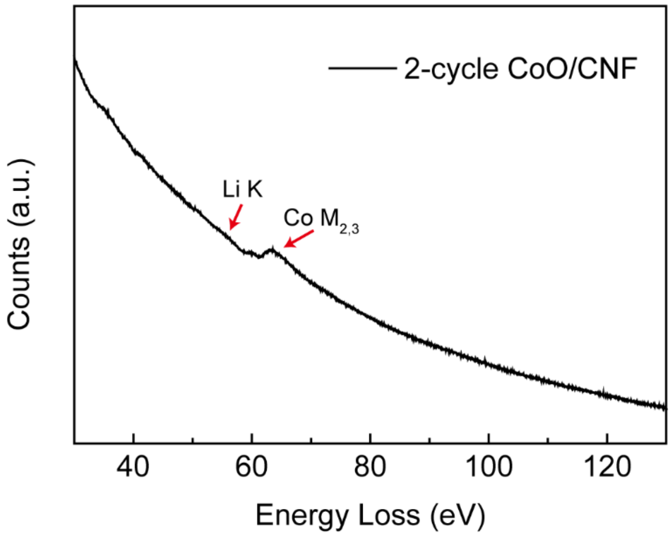

Supplementary Figure 10. Electron energy loss spectroscopy (EELS) of 2-cycle CoO/CNF. No observable Li K edge is shown, indicating that the amount of residual Li is under the detection limit of EELS.

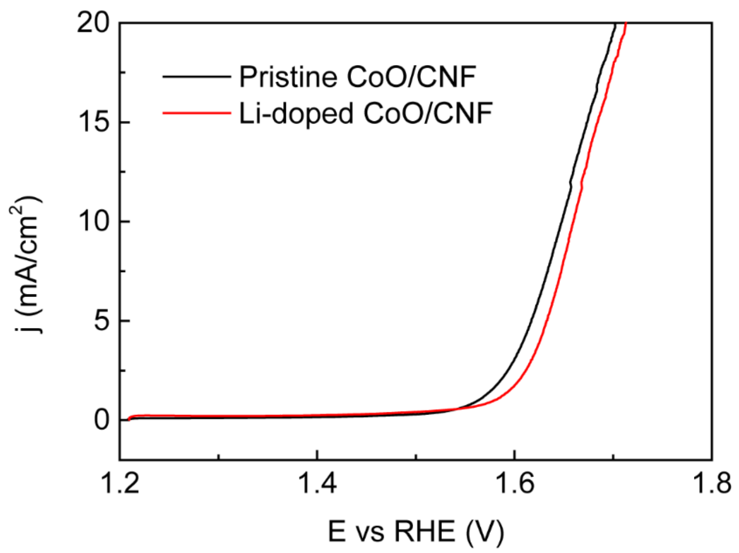

Supplementary Figure 11. OER activities of pristine and Li-doped CoO/CNF. To shed light on how Li doping influences the catalytic activities, we intentionally doped CoO/CNF with Li by charging the electrode to 1 V vs Li^+/Li (right above the conversion reaction plateau, Li to Co ratio was determined to be 1 : 7 by ICP-MS). The OER performance shows a slightly decay compared with pristine CoO/CNF, indicating that Li doping does not contribute to the improvement in OER performance.

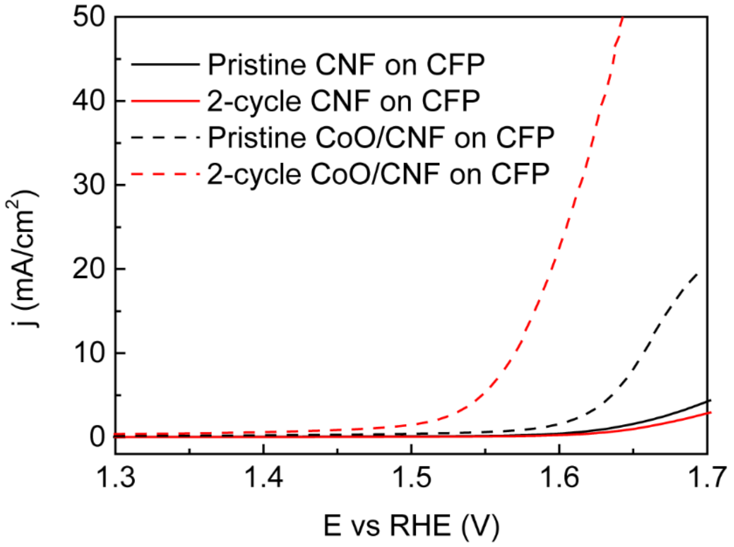

Supplementary Figure 12. OER background testing of pristine and 2-cycle CoO/CNF loaded on CFP. The backgrounds are negligible compared with pristine and 2-cycle CoO/CNF catalysts.

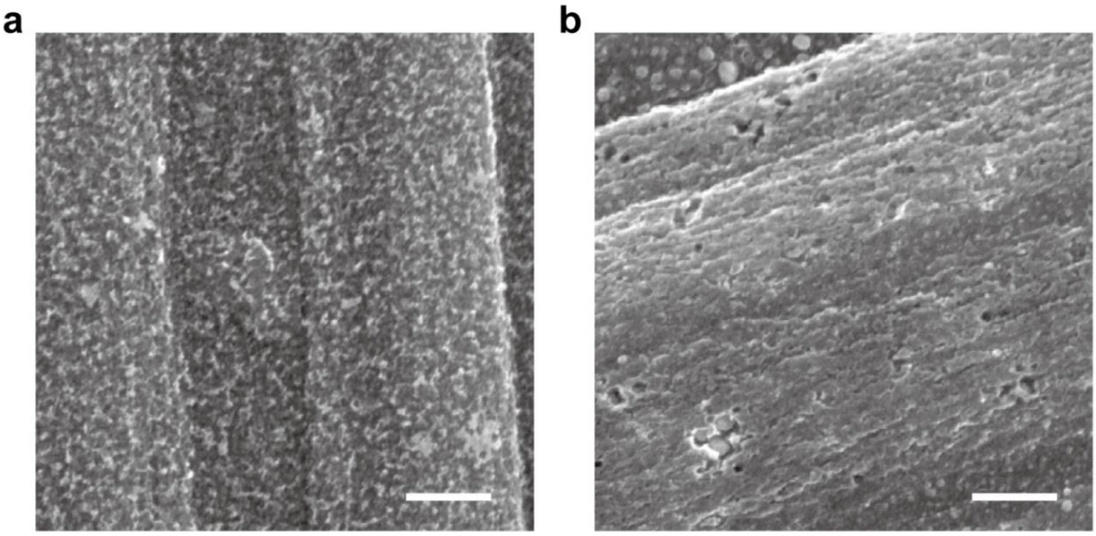

Supplementary Figure 13. SEM images of pristine (**a**) and 2-cycle (**b**) CoO/CFP. Scale bars: 200 nm.

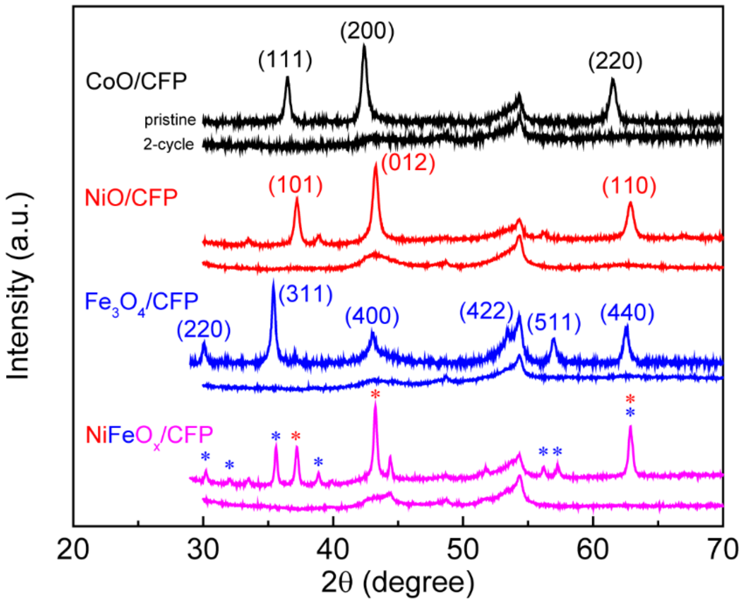

Supplementary Figure 14. XRD spectra of pristine and 2-cycle CoO/CFP, NiO/CFP, Fe_3O_4/CFP, and $NiFeO_x$/CFP. The resulted Fe_3O_4 phase suggests that under the synthesis condition Fe_3O_4 is more stable than Fe_2O_3. The $NiFeO_x$/CFP is actually a mixture of NiO and Fe_2O_3. The present of NiO might help to prevent Fe_2O_3 to be reduced to Fe_3O_4. After 2 battery cycles all of the patterns of TMOs become featureless, indicating the significantly reduced particle size which is below the coherence length of the X-ray.

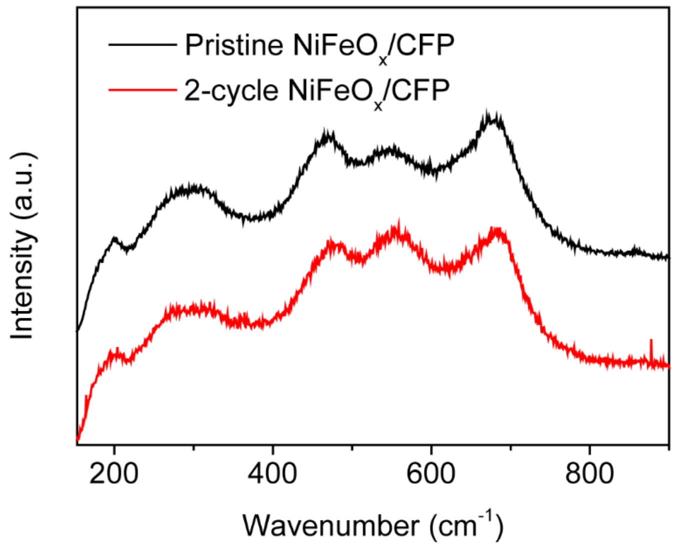

Supplementary Figure 15. Raman spectra of pristine and 2-cycle $NiFeO_x$/CFP. There are no significant changes in the peaks after 2 battery cycles, consistent with the observation on CoO/CNF in Supplementary Fig. 5

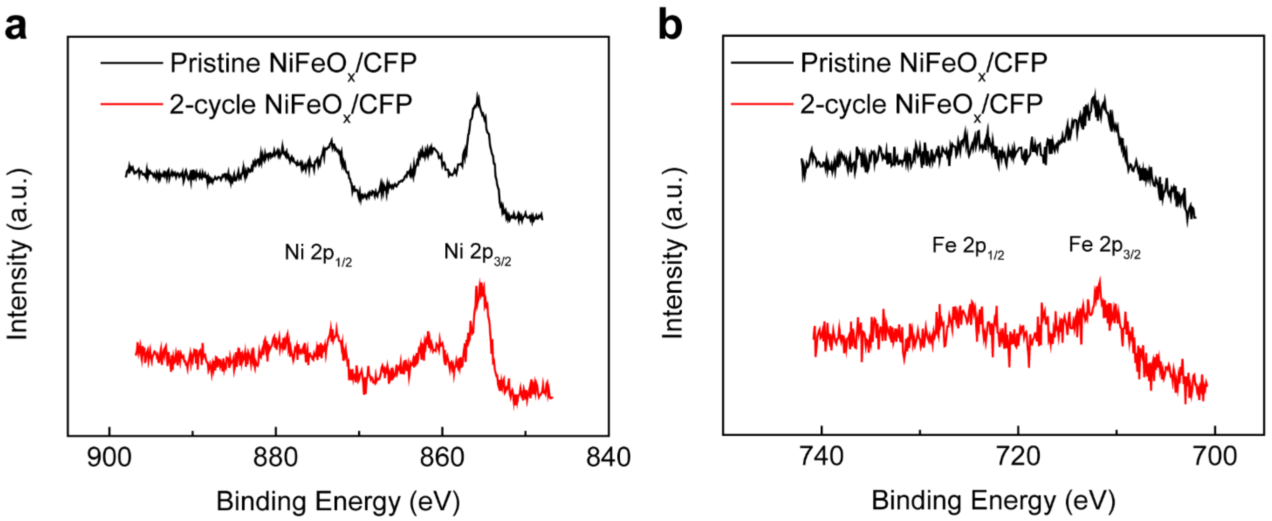

Supplementary Figure 16. X-ray photoelectron spectroscopy (XPS) of pristine and 2-cycle $NiFeO_x$/CFP. (**a**) Pristine and 2-cycle $NiFeO_x$/CFP show similar Ni 2p regions with both Ni $2p_{3/2}$ peaks located at ~ 855.6 eV. (**b**) Pristine and 2-cycle $NiFeO_x$/CFP show similar Fe 2p regions with both Fe $2p_{3/2}$ peaks located at ~ 711.9 eV.

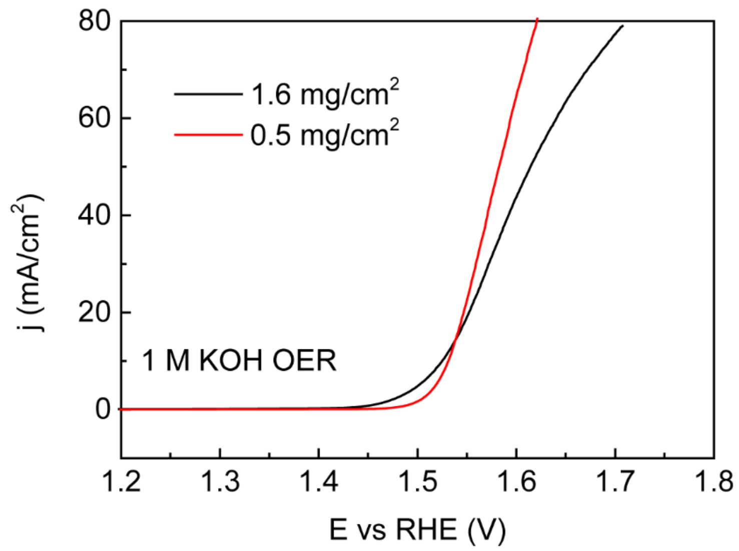

Supplementary Figure 17. The comparison of 0.5 mg/cm^2 and 1.6 mg/cm^2 Ir/C loadings for OER. Even though the large loading shows a lower onset potential, its activity is surpassed by the 0.5 mg/cm^2 loading when the current becomes significant. We suspect that the reason may due to the carbon additives in the commercialized Ir/C catalyst. It is understood that the carbon nanoparticles have hydrophobic nature, therefore too much loading may result in a hydrophobic surface on the electrode. This will hamper the contact between the electrode and electrolyte, and at the same time make it difficult for the gas products to release, resulting in degraded performance under high current density.

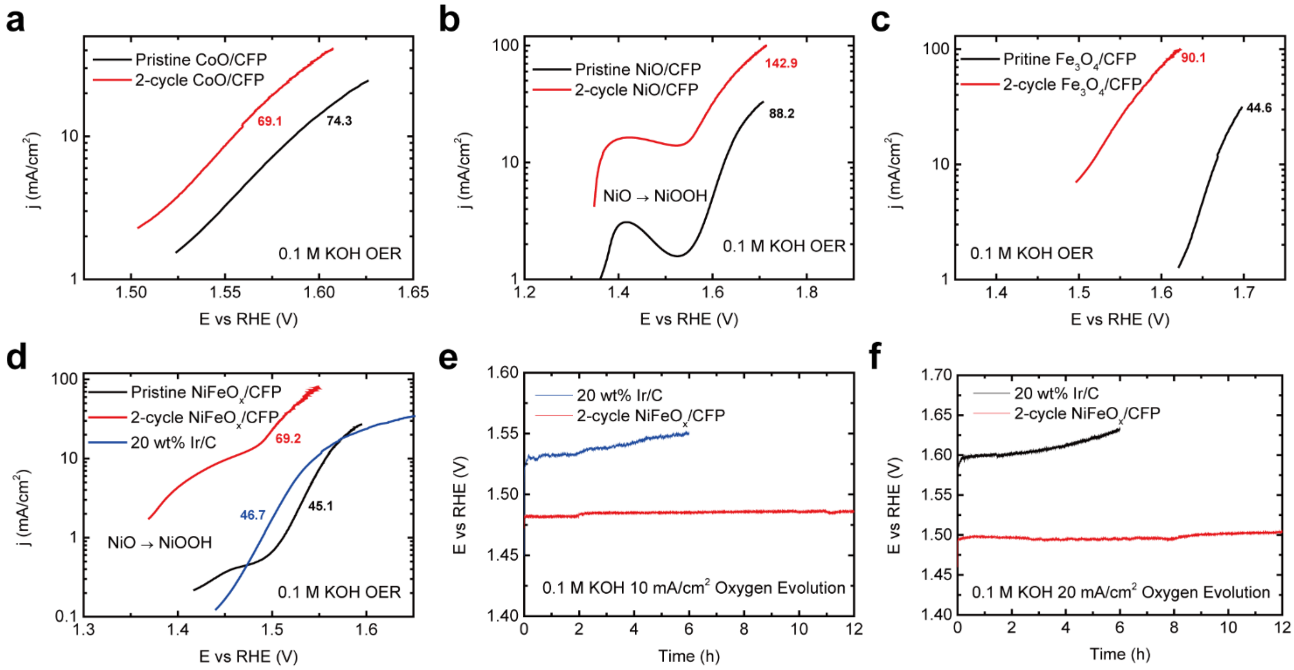

Supplementary Figure 18. The Tafel plots and stability testing of TMO/CFP samples. (**a**) The Tafel plots of pristine and 2-cycle CoO/CFP. (**b**) The Tafel plots of pristine and 2-cycle NiO/CFP. Since the oxidation peak overlaps with the OER onset currents, the Tafel slopes we obtained may be influenced by the bubble-releasing which gives out overestimated values. (**c**) The Tafel plots of Fe_3O_4/CFP. (**d**) The Tafel plots of pristine and 2-cycle $NiFeO_x$/CFP and Ir/C benchmark. (**e**), (**f**) Stability testing of 2-cycle $NiFeO_x$/CFP under constant current operation for over 12 hours.

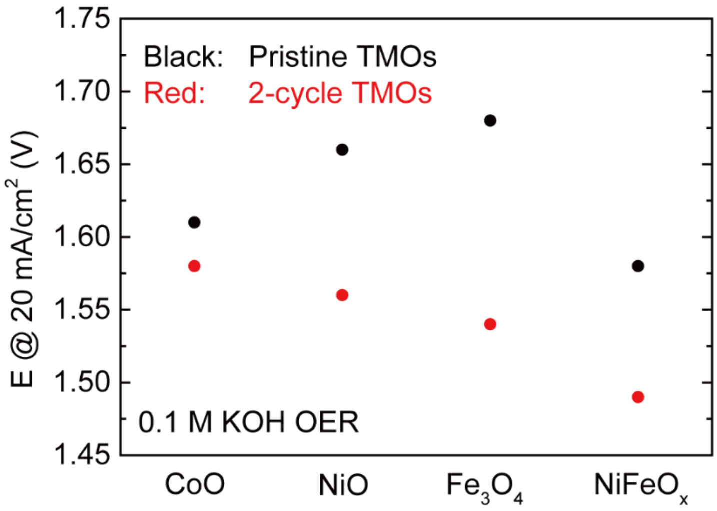

Supplementary Figure 19. Overpotentials of pristine and 2-cycle TMO/CFP catalysts to achieve 20 mA/cm^2 OER current. The improvements are significant after the galvanostatic cycling processes.

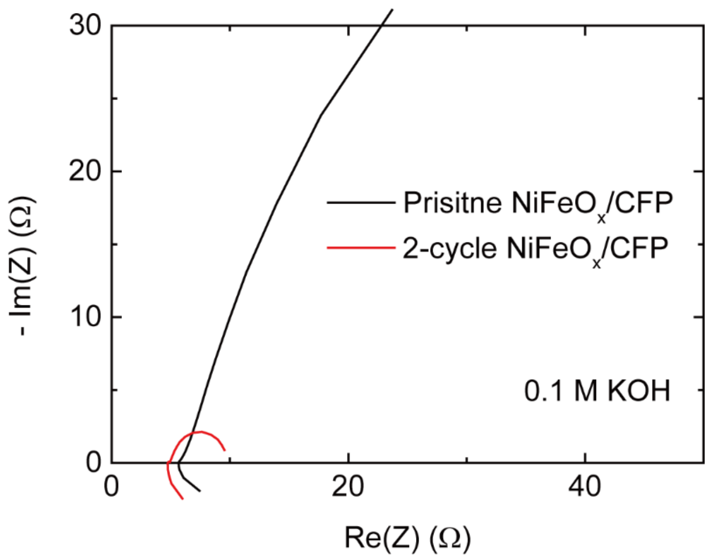

Supplementary Figure 20. Impedance spectra of pristine and 2-cycle $NiFeO_x$/CFP at 1.5 V vs RHE. The charge transfer impedance is significantly reduced after the galvanostatic cycling process.

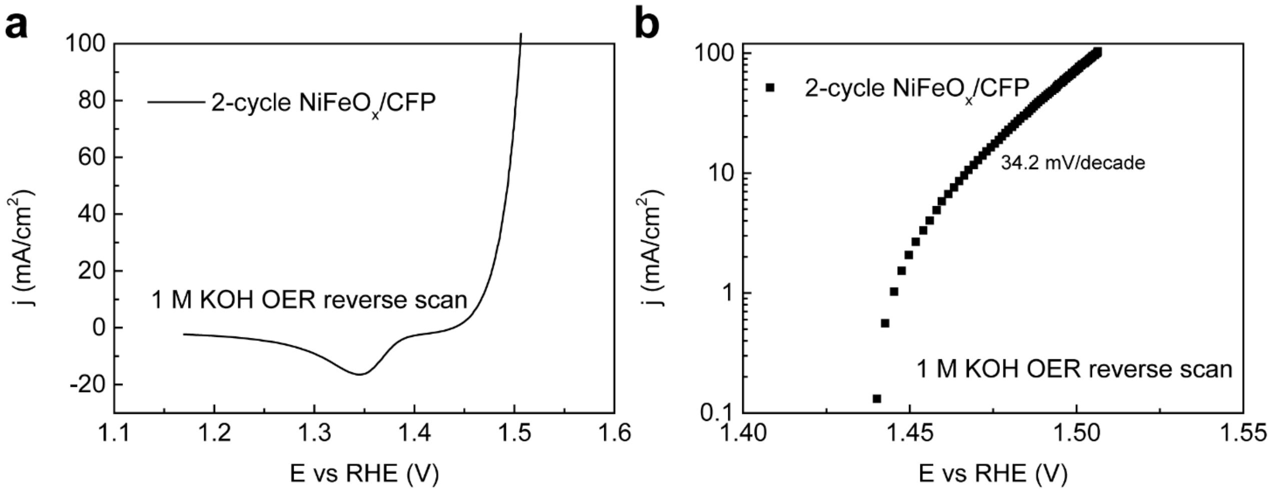

Supplementary Figure 21. Linear sweep voltammogram of 2-cycle $NiFeO_x$/CFP scanning from positive to negative potentials (reverse scan). (**a**) OER polarization of 2-cycle $NiFeO_x$/CFP by reverse scanning. (**b**) The corresponding Tafel plots of the reverse scanning OER polarization. The Tafel slope we obtained from the reverse scanning is 34.2 mV/decade, very close to what we previously obtained by the forward scanning in Fig. 3f.

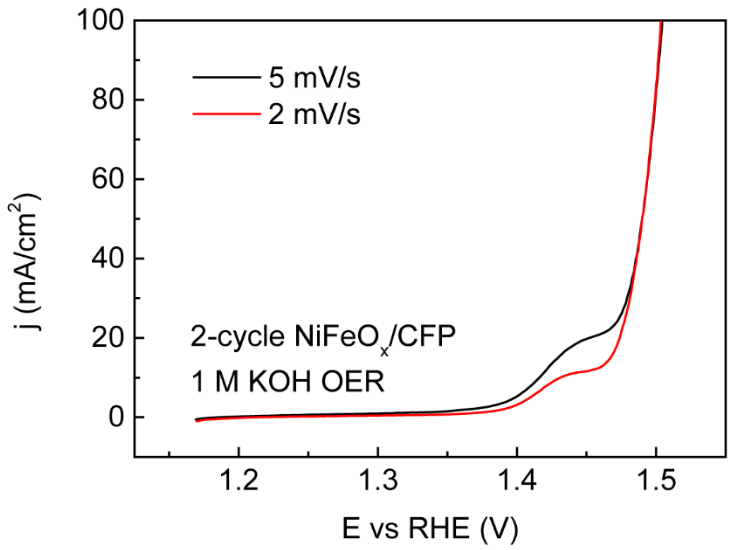

Supplementary Figure 22. OER polarizations of 2-cycle $NiFeO_x$/CFP under different scanning rates. The two polarization curves closely overlap within the OER range, suggesting that both rates are slow enough to reach the steady state for Tafel analysis. Note that a higher scanning rate will result in a larger redox peak of only catalyst particles which is reflected in the larger peak of 5 mV/s below.

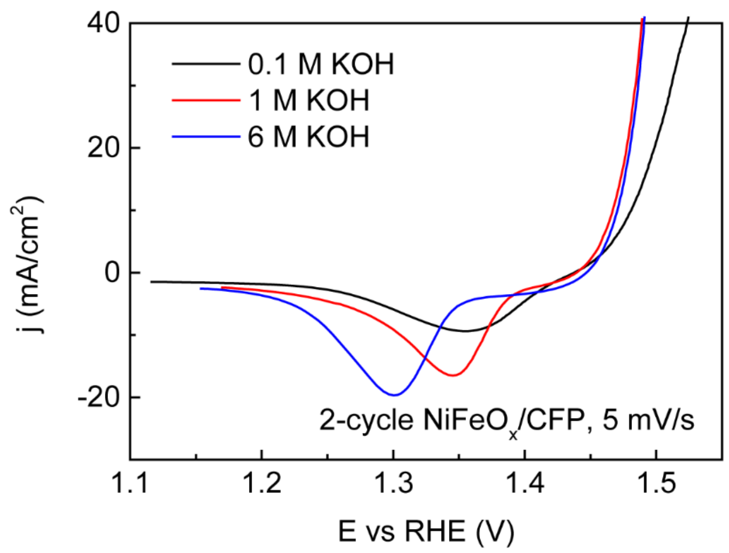

Supplementary Figure 23. OER polarizations of 2-cycle $NiFeO_x$/CFP in 0.1 M, 1 M, and 6 M KOH by reverse scanning. The areas of the reduction peaks should be equal to the oxidation peaks since this redox is reversible. In 1 M KOH the peak area is significantly larger than that in 0.1 M KOH, suggesting a deeper oxidation depth on the catalyst. In 6 M KOH the peak position is shifted from 1 M and the area is again increased. It is concluded that in more concentrated KOH the oxidation process can go deeper on the surface of the $NiFeO_x$ catalyst.

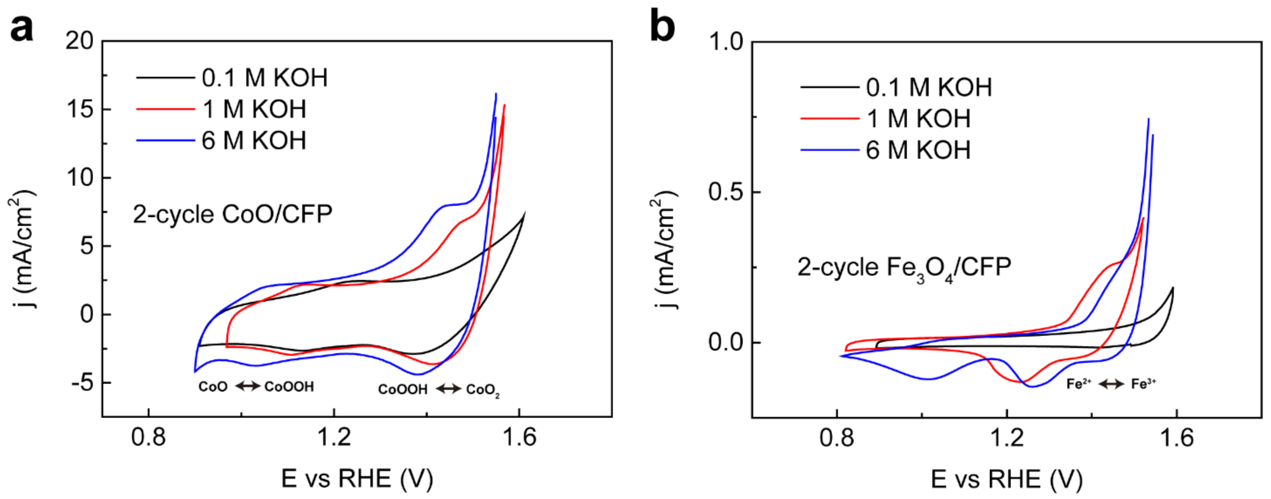

Supplementary Figure 24. Cyclic voltammograms (CVs) of 2-cycle CoO/CFP (**a**) and Fe_3O_4/CFP (**b**) at a slow scan rate of 5 mV/s in different concentrated KOH solutions. Different oxidation peaks are observed and assigned[2,3].

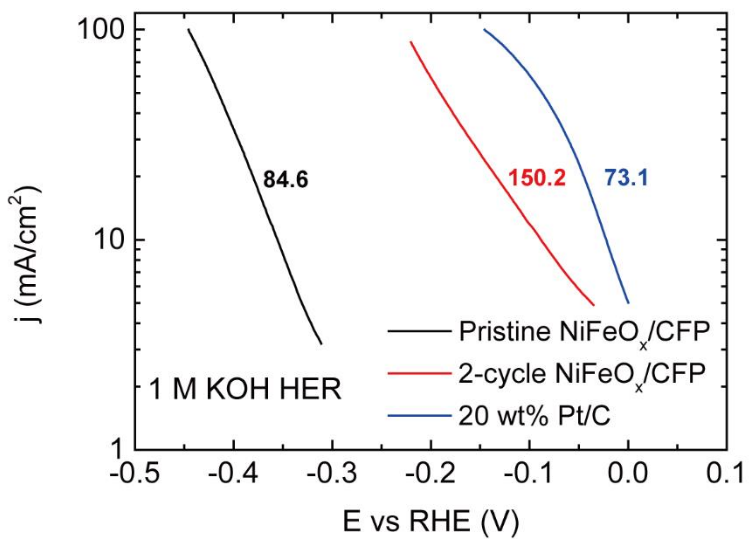

Supplementary Figure 25. Tafel plots of pristine and 2-cycle $NiFeO_x$/CFP and Pt/C benchmark for HER.

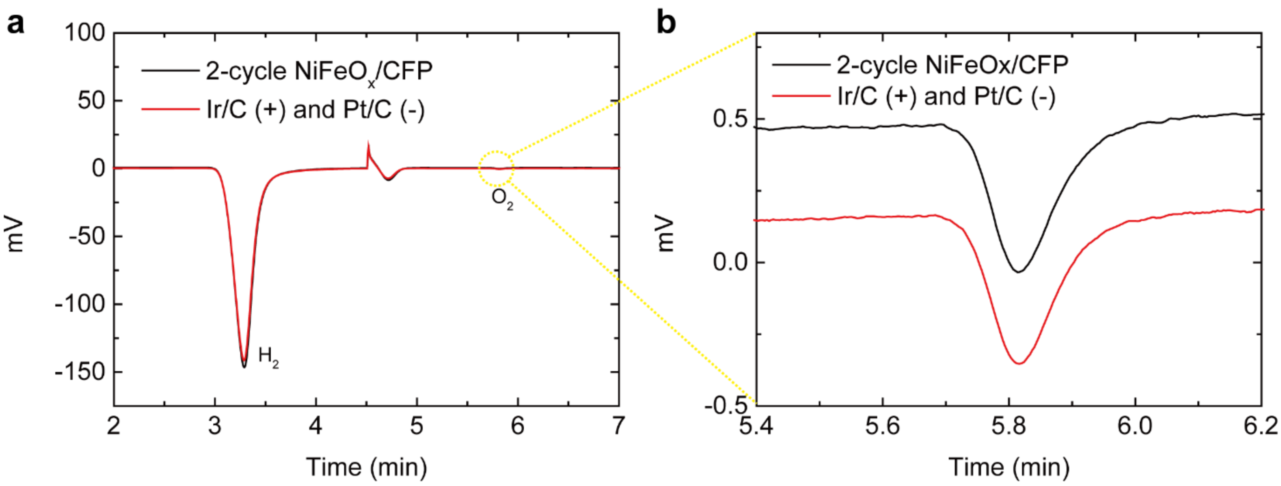

Supplementary Figure 26. Gas chromatography measurements of H_2 and O_2 produced by 2-cycle $NiFeO_x$/CFP bifunctional catalyst and Ir and Pt benchmark combination. The catalysts were first operated under 50 mA/cm^2 constant current for over 2 hours with Ar flow (5 sccm) as the carrier gas to saturate the whole testing system. The sampling process was taken after that. The H_2 and O_2 peaks have almost the same area between 2-cycle $NiFeO_x$/CFP and benchmark combination, indicating the high faradic efficiencies of both H_2 and O_2 produced by 2-cycle $NiFeO_x$/CFP. In addition, we also use standard gases with different H_2 concentrations to calibrate the H_2 production efficiency. The calculated FE of H_2 is 97 %.

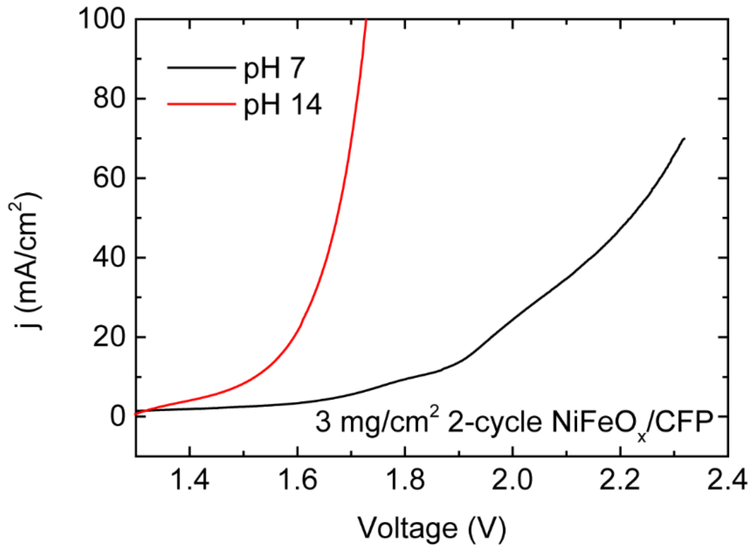

Supplementary Figure 27. OER polarizations of 2-cycle NiFeOx/CFP in different pH solutions. The pH 7 buffer is 1 M K_2HPO_4/KH_2PO_4. The water-splitting activity in alkaline solution is much better than that in neutral.

Supplementary References:

1 Johnson, S. H. *et al.* Co@CoO@Au core-multi-shell nanocrystals. *Journal of Materials Chemistry* **20**, 439-443 (2010).

2 Chen, K., Chen, X. & Xue, D. Hydrothermal route to crystallization of FeOOH nanorods via $FeCl_3 \cdot 6H_2O$: effect of Fe^{3+} concentration on pseudocapacitance of iron-based materials. *CrystEngComm* **17**, 1906-1910 (2015).

3 Li, J., Yang, M., Wei, J. & Zhou, Z. Preparation and electrochemical performances of doughnut-like $Ni(OH)_2$-$Co(OH)_2$ composites as pseudocapacitor materials. *Nanoscale* **4**, 4498-4503 (2012).
